# QuickStats

**Published:** 2014-09-12

**Authors:** FROM THE NATIONAL CENTER FOR HEALTH STATISTICS

**Figure:**
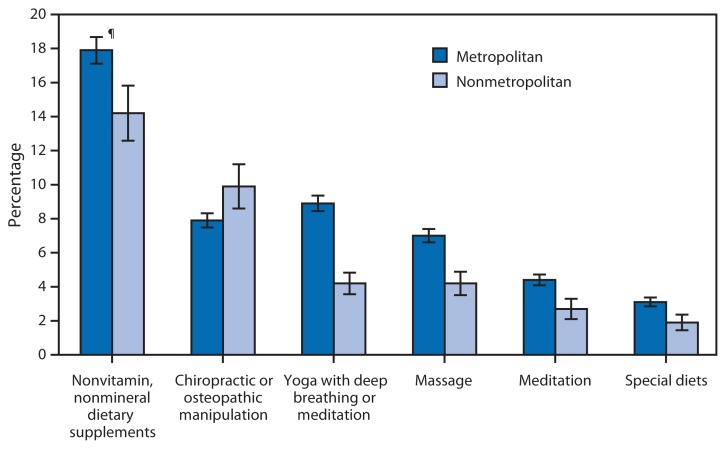
Percentage of Adults Who Used Selected Complementary Health Approaches* in the Preceding 12 Months, by Metropolitan Status of Residence^†^ — National Health Interview Survey,^§^ United States, 2012 * Based on the six most commonly used complementary health approaches among U.S. adults in 2012. ^†^ Based on the household residence location. Metropolitan is located within a metropolitan statistical area, defined as a county or group of contiguous counties that contains at least one urbanized area of ≥50,000 population. Surrounding counties with strong economic ties to the urbanized area also are included. Nonmetropolitan areas do not include a large urbanized area and are generally thought of as more rural. ^§^ Estimates are based on household interviews of a sample of the civilian noninstitutionalized U.S. population. ^¶^ 95% confidence interval.

During 2012, the percentages of U.S. adults aged ≥18 years who used nonvitamin, nonmineral dietary supplements, yoga, massage, meditation, and special diets were higher in metropolitan areas than in nonmetropolitan areas. A greater proportion of adults in nonmetropolitan areas used chiropractic or osteopathic manipulation (9.9%) compared with those in metropolitan areas (7.9%). In both metropolitan and nonmetropolitan areas, dietary supplements had the highest percentage of use (17.9% in metropolitan; 14.2% in nonmetropolitan), and special diets had the lowest percentage of use (3.1% in metropolitan; 1.9% in nonmetropolitan).

**Source:** National Health Interview Survey, 2012. Available at http://www.cdc.gov/nchs/nhis.htm.

**Reported by:** Lindsey Jones, MPH, izf4@cdc.gov, 301-458-4548; Tainya C. Clarke, PhD; Patricia Barnes, MA.

